# Transorbital-penetrating intracranial injury due to a homemade metal arrow: A case report

**DOI:** 10.1016/j.amsu.2020.07.049

**Published:** 2020-07-28

**Authors:** Eko Prasetyo, Maximillian Christian Oley, Vera Sumual, Muhammad Faruk

**Affiliations:** a Division of Neurosurgery, Department of Surgery, Faculty of Medicine, University Sam Ratulangi, Manado, Indonesia; b Division of Neurosurgery, Department of Surgery, R. D. Kandou Hospital, Manado, Indonesia; c Department of Neuroscience, Siloam Hospital Manado, Manado, Indonesia; d Department of Ophthalmology, Faculty of Medicine, University Sam Ratulangi, Manado, Indonesia; e Department of Ophthalmology, R. D. Kandou Hospital, Manado, Indonesia; f Department of Surgery, Faculty of Medicine, Hasanuddin University, Makassar, Indonesia

**Keywords:** Transorbital, Penetrating, Intracranial injury, Metal arrow, Case report.

## Abstract

A transorbital-penetrating intracranial injury (TOPI) is an unusual traumatic brain injury. This rare injury has the potential to result in serious and fatal brain damage with a high mortality rate and requires prompt multidisciplinary surgical intervention. Here, we describe an interesting case in which a patient who presented with accidental penetrating injuries of the brain was found to have a transorbital-penetrating intracranial injury (TOPI). We chose an anterior approach to the foreign body above the entrance wound for removal in a retrograde manner with fluoroscopic guidance. The patient remained well with no complications and was discharged on postoperative day 10. Reasonable diagnostic imaging, surgical planning, and careful post-surgery management can increase patients successful outcomes.

## Highlights

• A transorbital-penetrating intracranial injury (TOPI) is an uncommon traumatic brain injury.
• TOPIs have the potential to result in serious brain damage with a high mortality rate.
• An interesting case of accidental TOPI that was treated successfully with fluoroscopic guidance is presented.

## 1 Introduction

Accidental penetrating injuries of the brain are relatively uncommon [1]. Although a transorbital-penetrating intracranial injury (TOPI) is uncommon, its potency causes severe brain injury and is related to high mortality rates [2,3]. This injury, accounting for 4.5% of all orbital abnormalities, represents 0.04% of all head injuries, with penetrating head-trauma incidents in adults at about 24% and children 45% [4].

TOPI, while uncommon, can result in serious damage of the orbital and brain structures and even death if not promptly treated [5,6]. This unusual injury is associated with intracranial complications such as brain abscess, meningitis, cerebrospinal-fluid (CSF) leakage, hemorrhage, neurological deficits, and mortality [6,7]. The reported prevalence of vascular complications following TOPI is as high as 50%, and this complication can be life-threatening [8]. The mortality rate for TOPIs is 33% in cases of timely surgical treatment and increases to 53% in cases where surgery is delayed [7,9]. Surgery is the major strategy for the treatment of TOPIs, and surgical indications include a retained foreign body, CSF leakage, fracture displacement, intracranial hemorrhage, and vascular injury. The surgical approach to orbital cranial foreign bodies can be performed in two ways depending on location: the extra-orbital, such as transcranial, or the transorbital approach [10]. The present study reports the uncommon case of a male suffering a TOPI in accordance with the Surgical Case Report (SCARE) 2018 guidelines [11].

## 2 Case presentation

A 28-year-old male came to our Emergency Department with vision loss in his right eye following an accident that occurred an hour earlier at his workplace. The patient is a farmer who plans to hunt eels in rice fields. He was using a 4.5mm air rifle with projectiles consisting of metal arrows made from motorcycle wheel spokes. When the gun jammed, he checked it through the gun barrel, and the accident happened when the rifle was accidentally triggered. A metal arrow entered his head and penetrated his right infraorbital region.

On arrival, his vital signs were normal limit and his mental status was alert. Primary and secondary surveys did not reveal additional injuries, and no significant medical history or drugs, tobacco, or alcohol abuse was recorded. The patient reported having a headache but no nausea, vomiting, or convulsions. In addition, he exhibited normal body temperature, a Glasgow Coma Scale score of 15 points, clear consciousness and speech, and cooperation during the physical examination. The muscular strength and tension of the limbs were normal. Bilateral Babinski signs were not induced. He was given a preoperative test immediately after admission, as well as an intravenous broad-spectrum antibiotic, anti-tetanus injection, and anti-convulsant medications. Blood analyses and other biochemical parameters were within normal limits.

**Fig. 1:**
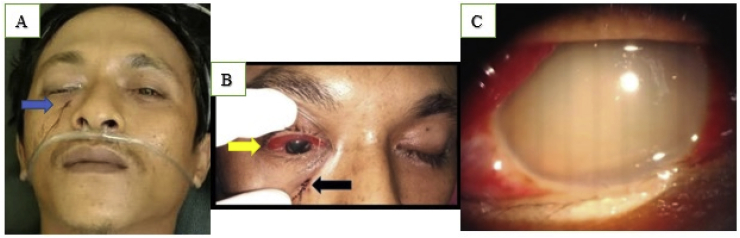
A). Entry site of the foreign body (arrow). B). Minimal ecchymosis and hyphemia (yellow arrow) and the penetration wound in Zone 3C of the Turbin pattern (black arrow). C). a vitreous hemorrhage in the patient's right eye. (For interpretation of the references to colour in this figure legend, the reader is referred to the Web version of this article.)

**Fig. 2:**
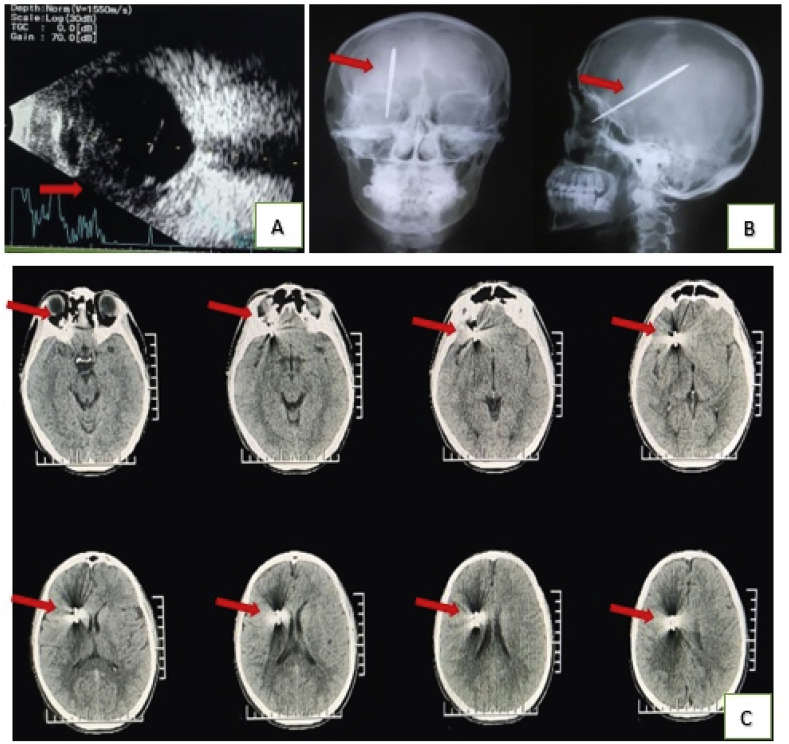
Radiological Findings: A). Ocular ultrasonography presenting a discontinuity of the inferior wall of the eyeball (arrows). B). Plain skull x-ray (anteroposterior and lateral view) showing the homemade metal arrow penetrating the right orbital roof and traversing to the ipsilateral parietal lobe (arrows). C). Non-contrast CT of the brain depicting the metal arrow's trajectory posteriorly through the right orbital roof (arrows).

Local examination revealed a perforating injury at the entry location of the foreign body and penetration below the right infraorbital margin measuring 0.5 × 0.5 cm in size with bleeding (Fig. 1A). His right pupil was dilated and not reactive to light, and eye movement was limited. Only minimal ecchymosis and hyphemia were seen in the anterior chamber of the right eye. There was a penetrating wound on the right inferior medial eyelid or Zone 3C of the Turbin pattern measuring 1 × 0.5 cm (Fig. 1B). Using a slit lamp, we found a vitreous hemorrhage (Fig. 1C), and ocular ultrasound revealed a discontinuity of the inferior wall of the eyeball (Fig. 2A).

**Fig. 3:**
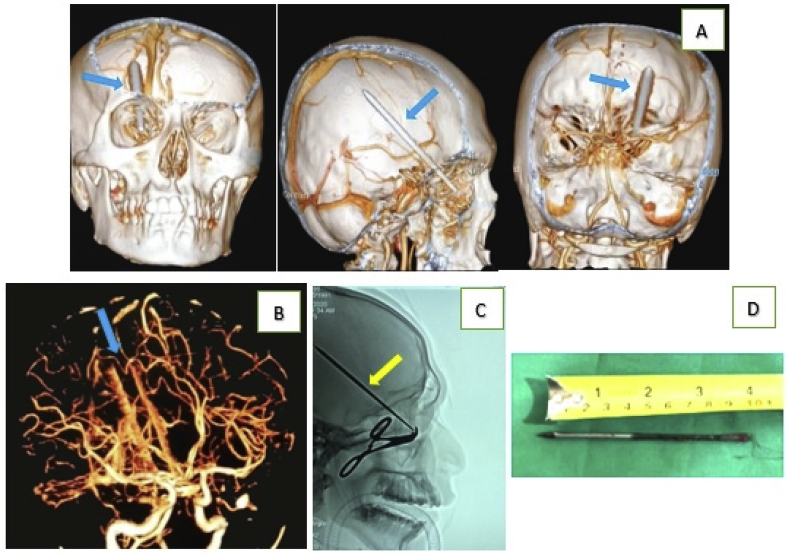
A). 3D CT (anterior, lateral, and posterior) showing the passage of the arrow through the right orbital roof into the ipsilateral cranium (arrows). B). CT angiography documented that there was no injury to the main vascular brain structures (arrows). C). C-arm guidance to remove the arrow (arrows). D). A metal arrow 10 cm in length.

A plain skull radiograph showed that a metallic foreign body that looked like an arrow had penetrated above the right orbital roof (Fig. 2B). The tip of the foreign object was in the right parietal lobe as revealed by non-contrast computerized tomography (CT) (Fig. 2C) and a three-dimensional CT (3D CT) of his brain (Fig. 3A). No lesions of the major cerebral vessels were noted on non-contrast CT angiography (CTA) (Fig. 3B).

As there was no evidence of vascular injury and the neurosurgeon recommended anterior orbital rather than transcranial surgery, it was considered reasonable and safe to remove the foreign body anteriorly. Subsequently, with preparing and planning surgery having established a multidisciplinary approached, the patient was transferred to the operating room 2 h after arriving in the Emergency Department. We (the neurosurgeon and ophthalmologist) performed retrograde removal through the penetration wound with C-arm radiography fluoroscopic guidance (Fig. 3C). We used fluoroscopic guidance during the surgery to determine the precise location of the base of the arrow through the penetration or entrance wound and to perform retrograde removal gently. There was a minimal amount of brain tissue accompanying the arrow from the entry wound without CSF leakage. The length of the homemade metal arrow was 10 cm (Fig. 3D). Following this procedure, we irrigated the wound with a standard 0.9% saline solution containing antibiotics to eliminate debris and control bleeding in the entrance wound and wound track as far as could be attained.

**Fig. 4:**
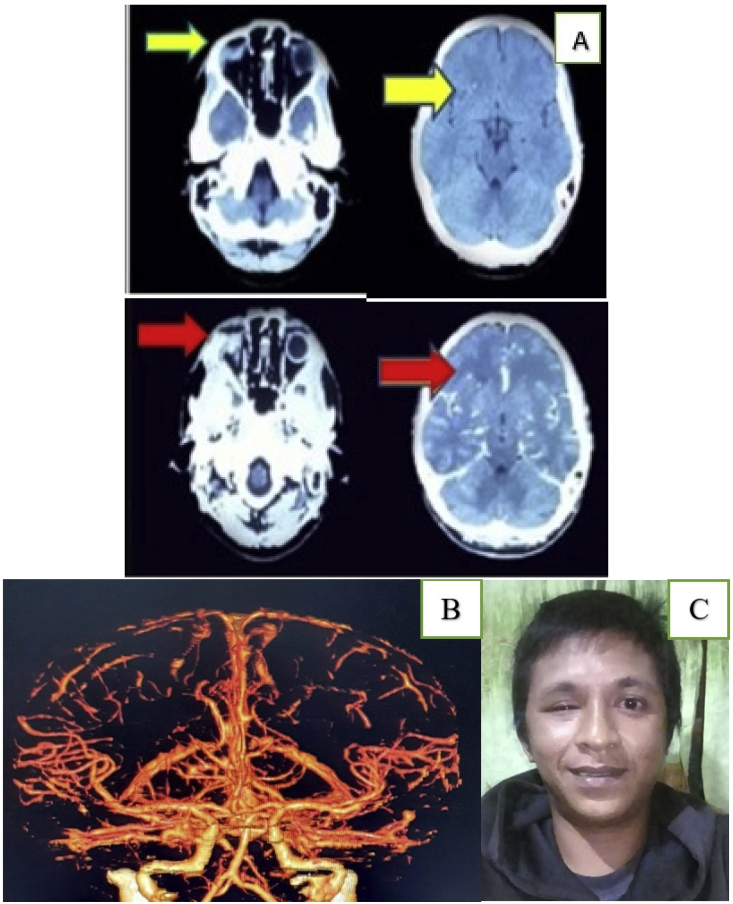
The post-operative evaluation six months later A). A CT brain scan with (red arrow) and without contrast (yellow arrow) presented a slight ischemia in the foreign-body track (right frontal lobe) and a globe rupture on the right eye. B). A CTA does not appear to show abnormal vascular brain structures. C). Enophthalmos in the patient's right eye. (For interpretation of the references to colour in this figure legend, the reader is referred to the Web version of this article.)

Post-surgery, the patient was admitted to the critical care unit, where broad-spectrum antibiotics and anticonvulsant drugs were administered for seven days. His intracranial pressure (ICP) was monitored by the neurosurgeon in the postoperative period for two days and was within normal limits. The patient fully recovered and was discharged on postoperative day 10. The long-term, post-operative evaluation six months later, as well as the surgical wound and the patient's general condition, were satisfactory. The CT brain scan with and without contrast showed a slight ischemia on the right-frontal lobe and a globe rupture on the right eye (Fig. 4A). The CTA detected no abnormal vascular brain structure (Fig. 4B). Meanwhile, the patient's vision had not changed, with enophthalmus in the right eye (Fig. 4C).

## 3 Discussion

Transorbital-penetration injuries of the skull and brain are quite rare, but TOPIs can cause significant ophthalmic and neurological disabilities [6]. The presentation of a TOPI depends on size, orientation, type of object, and depth of penetration. Because the orbit is a rectangular pyramid, a foreign body can penetrate the superior, inferior, lateral, or medial edges [3]. The orbital bones are funnel-shaped anatomical structures with sclera that tend to be weak. The superior orbital fissure lies at the apex and provides a passage for cranial nerves such as III, IV, and VI into the middle cranial fossa [10]. Foreign bodies more than 5 cm long can penetrate the cavity of the skull through the orbital cavity [12]. The pathogenic foreign body reported in the present case report was longer than 5 cm and penetrated the brain.

Foreign bodies reach the brain via three main routes: the optic canal, the inferior orbital fissure, and the orbital roof [13]. The fragile bone and thinner parts of the orbital roof make it the most frequent route and foreign bodies can reach the meninges, brain parenchyma, and vascular structures, which can damage the frontal lobe [10]. The most-common penetrating pathway through the orbital roof is due to the fragile structure of the superior orbital plates of the frontal bone, leading to frontal lobe contusion cases [5].

Turbin et al. found patterns of transorbital intracranial injury in 38 cases; he divides the orbital surface into four distinct zones [14]. Our case is categorized as Zone 3C, the inferior medial region of the right orbit (Fig. 1B). These four routes for TOPIs are related to various locations of the central nervous system (CNS) injury. Balasubramanian et al. classified TOPIs based on the anatomy of the orbital bones and their associated damage [15]. An analysis of injury patterns is vital to assist surgeons in adjusting management and surgical approaches and in anticipating the location and potential types of intracranial complications associated with foreign-body penetration [14,15].

In the case being reported, we found a discontinuity of the inferior wall of the eyeball on ocular ultrasound (Fig. 2A). Ultrasonography is usually used to detect posterior-segment foreign bodies; however, its operator-dependent nature limits the use of this technology. A highly skilled ultra-sonographer is very helpful when identifying the anterior segment [16,17].

A plain skull radiograph confirmed that the metal arrow had penetrated the right orbital roof, and the metal tip was lodged in the right parietal lobe (Fig. 2B). An evaluation of the radiological examination revealed that the plain skull x-ray was insufficient; in fact, when used as a first-line modality, such x-rays fail to detect deformities in more than 50% of cases.

In evaluating cases of TOPI with the use of plain skull x-rays, it has been found that the plain skull x-ray has a very high failure rate for detecting abscesses, retained foreign bodies (wood, plastic, and glass) and fractures [14]. In this case, a plain skull x-ray showed that the metal arrow had penetrated the right orbital roof and traversed to the ipsilateral parietal lobe (Fig. 2B). The plain skull x-ray played a vital role in the detection of the design at the tip of this metal arrow and whether the metal arrow's tip was jagged or had a fishhook pattern, which allowed retrograde removal to protect more extensive neurovascular damage.

Non-contrast CT is considered the “first line,” “central,” “key,” “optimal” or “mandatory” imaging modality for initial radiological assessment [5,10]. CT should be performed as soon as possible because it can identify any foreign bodies that remain, lesion extension, localization of foreign bodies and bone fragments, hematoma, penetration pathway, and other related lesions; it can also provide useful information for planning surgical procedures [18]. Our patient underwent a 3D CT for a detailed analysis of the bony pathology image, position, and trajectory of the retained foreign body. The complete structural anomaly encountered by the surgeon is not fully explained in planar reconstruction. The surgeon must have a three-dimensional image of the patient's skull abnormalities as a useful adjunct to surgical planning [15,19]. In this case, CT (Fig. 2C) and 3D CT (Fig. 3A) revealed a foreign body along the path of the metal arrow passing through the roof of the right orbital toward the ipsilateral parietal lobe.

The Schreckinger algorithm [20] and the Balasubramanian classification [15] showed that CTA should be performed to investigate the cerebrovascular injuries either by the location or trajectory of the foreign body after an intracranial penetrating trauma. CTA is accurate in detecting most traumatic intracranial aneurysms, dissections, and occlusions or for revealing the location of hematomas [7,[21], [22], [23]]. In this case, CTA demonstrated that no discernible injury to the surrounding middle cerebral artery (MCA) branches was observed (Fig. 3B).

The main goal of treatment for TOPIs is the prevention of infections, ranging from soft tissue and orbital to cerebrovascular structures [23,24]. This type of injury is part of a penetrating brain injury (PBI). The risk of local wound infections, meningitis, ventriculitis or cerebral abscess is particularly high among PBI patients because of the presence of contaminated foreign bodies (organic or not organic material), skin, hair, and bone fragments driven into the brain tissue along the wound track [25,26]. The orbital structures and soft tissue infection surrounding the projectile track was the initial source of the infection process in the TOPI case of an increased risk of infection by CSF leakage [27]. Staphylococcus aureus is the most frequently associated organism. Nevertheless, gram negative bacteria is also frequently the cause of intracranial infections following PBIs [26]. In the literature, the commonly reported pathogens for abscess formation or meningitis are streptococcus and staphylococcus [14]. There is considerable variation in the preference for an antibiotic regimen for prophylaxis in PBI patients. Cephalosporins, however, are the most-often used antibiotics [25,28]. Esposito and Walker recommended the use of the intravenous administration of ceftriaxone, vancomycin, and metronidazole for a minimum 6 weeks for PBI patients. The recommendation is that this regimen should be initiated as soon as possible after the injury and continued for 5 days postoperatively [25]. Based on several studies, Kazim and his colleagues recommended that antibiotic prophylaxis be maintained for at least 7–14 days [26]. The duration of antibiotic treatment varies, and other surveys in which cephalosporins were the most-common choice of antibiotic recommended treatment duration that ranged from 1 to more than 10 days [28].

The risk of post-traumatic epilepsy following a PBI is high, probably due to direct traumatic injury to the cerebral cortex with subsequent cerebral scarring [25,29]. The incidence of epilepsy in TOPI patients is approximately 30%–50% [27]. Therefore, prophylaxis antiepileptic drugs should be administered in the acute phase of injury to decrease the incidence of post-traumatic epilepsy. The postoperative prophylaxis use of sodium valproate is effective for preventing epilepsy [23]. Other studies have suggested a prophylaxis regimen such as phenytoin, carbamazepine, valproate, or phenobarbital. The use of anticonvulsants beyond the first 7 days of injury is generally not recommended [29].

Schreckinger et al. summarized the indications for surgical exposure succinctly in regard to retained foreign bodies, intracranial hematomas, displaced bone fractures, evidence of direct vessel injury, and the presence of dura mater defects [21]. The surgical approach to foreign bodies in the cranial orbitals can consist of two ways that depend on location: an extra-orbital, such as transcranial, or transorbital approach [12]. Appropriate radiological examinations with CT must be carefully reviewed in order to determine wise, safe treatment, and the need for caution regarding retained foreign bodies must also be communicated to the radiologist. If there is no evidence of vascular injury based on the results of a radiological examination, neurosurgeons will advocate an anterior orbital approach rather than transcranial surgery. It makes sense to remove penetrating transorbital-cranial foreign bodies anteriorly or using the transorbital technique [30].

In our case, the foreign body (a metal arrow) penetrated the right orbit's inferior medial. This penetration caused fractures of the orbital roof wall, and in the vertical plane, the metal arrow penetrated the orbital roof to reach the frontal lobe. There were no signs or evidence of potential intracranial vascular injury; only minimal hematomas around the wound and without CSF leakage were observed. We chose to take an anterior approach to remove the foreign object through the entrance wound to retrograde removal with fluoroscopic guidance and debride the wound's path to the intracranial distal section.

TOPIs, although uncommon, can result in serious damage of the orbital and brain structures, and may result in death if not promptly treated [5]. Lower-velocity objects produce a track of primary tissue damage, traversing a straight course and usually associating the injury with the bony and neurovascular structures in the path of the traversing foreign body leading to orbital and focal localized brain parenchymal injury [23]. The orbital roof is relatively thinner and more easily penetrated by a foreign body, which can travel into the cranial cavity causing extensive damage to the brain parenchyma as well as meningeal and vascular injuries [31].

Early complications of TOPIs include intracerebral hematoma, cerebral contusion, intraventricular hemorrhage, pneumocephalus, brainstem injury, and traumatic pseudoaneurysms known as carotid-cavernous fistulas [9]. The reported prevalence of intracranial vascular injury following a TOPI is as high as 50%, and this complication can be life-threatening [8]. Taylor and Peter reported a 25% vascular complication rate in a series of 66 patients with TOPIs [32]. There are three main types of vascular injuries following TOPIs: subarachnoid hemorrhage, traumatic intracranial aneurysm, and arterial dissection [8].

Infection is the most-fatal complication of TOPIs, with reported overall rate of 64–70% and a mortality rate of 14–57% [19]. The incidence of traumatic brain abscesses in the civilian population ranges from 2.5% to 10.9% of total brain abscesses. Penetration of the orbital roof allows for the inoculation of bacteria, posing a potentially serious complication. The retention of foreign bodies and bone fragments in the track of a TOPI may also be a main source of infection [24,33]. Meningitis, abscess, or empyema can appear days, weeks, or months after the traumatic injury [24]. Mortality in the extant literature has been reported to be due to infection and lack of optimal antimicrobial therapy [6,29,30]. In approximately 20% of patients, microbiological cultures of abscessed material remain sterile [33]. The surgical culture was negative in our patient.

Therefore, the mortality rate of TOPIs is 33% in cases where timely surgical treatment is performed, and this rate increases to 53% in cases of delayed surgery [7]. Regarding the selection of surgical timing, extremely early foreign-body removal may cause massive bleeding due to the change in the pressure gradient, while delayed surgery results in an increased risk of infection. The current recommendation is that surgery be performed within 12 hours [19,26].

## 4 Conclusion

We present the case of a patient with a TOPI resulting from an accident involving a homemade metal arrow. The outcome of TOPIs depends on the location of the penetration path, the degree of neurovascular damage, and neurological status. We emphasize the importance of radio-imaging diagnosis as a guide to selecting the appropriate surgical intervention, and a multidisciplinary approach is essential to ensure decreased patient disability and mortality rates.

## Provenance and peer review

Not commissioned, externally peer reviewed.

## Ethical approval

The study is exempt from ethical approval in our institution.

## Sources of funding

No funding or sponsorship.

## Author contribution

EP, MCO, VS, and MF researched the literature and wrote the manuscript. EP and VS operated on the patient and had an idea for this case report. EP, MCO, VS, and MF checked the manuscript and made corrections. EP and MCO provided the overall guidance and support. All the authors read and approved the final manuscript.

## Registration of research studies

None.

## Guarantor

Eko Prasetyo.

## Consent

Written informed consent was obtained from the patient for publication of this case report and accompanying images.

## Declaration of competing interest

The authors declare that they have no conflict of interests.
